# Developing an obstetric care screening tool to improve social support access for pregnant women: A prospective cohort study

**DOI:** 10.3389/fgwh.2022.921361

**Published:** 2023-02-01

**Authors:** Haruna Kawaguchi, Ryoji Shinohara, Yuka Akiyama, Megumi Kushima, Yoshio Matsuda, Marie Yoneyama, Tomomi Yamamoto, Zentaro Yamagata

**Affiliations:** ^1^ Department of Maternal-Fetal Medicine, Osaka Women's and Children's Hospital, Osaka, Japan; ^2^ Center for Birth Cohort Studies, University of Yamanashi, Yamanashi, Japan; ^3^ Department of Health Sciences, School of Medicine, University of Yamanashi, Yamanashi, Japan; ^4^ Department of Obstetrics, Toho Medical Clinic, Tokyo, Japan; ^5^ Postgraduate School of Healthcare, Division of Midwifery/ Innovative Nursing Practice, Tokyo Healthcare University, Tokyo, Japan; ^6^ Department of Nursing, International Catholic Hospital, Tokyo, Japan

**Keywords:** pregnant women, social support, interview sheet during pregnancy, screening tool, maternal mental health, child maltreatment

## Abstract

**Background:**

This study aimed to develop an efficient interview sheet during pregnancy and screening tool to identify pregnant women needing social support at obstetric institutions. Moreover, we investigate the perinatal factors associated with the Edinburgh postnatal depression scale (EPDS).

**Methods:**

This prospective cohort study was conducted at three medical institutions that commonly encounter cases with social issues. Pregnant women were assessed using an interview sheet at the first visit (*n* = 678), at approximately 28 weeks gestation (*n* = 495), 36 weeks gestation (*n* = 296), and the postpartum period (*n* = 822). We investigated the important items identified on the new screening questionnaire (NEW interview sheets) for women needing social support. The items on the interview sheet were scored by multiple linear regression analysis, and the cutoff values were calculated using the receiver operating characteristic curve. The association between perinatal factors and EPDS was assessed using multiple logistic regression analyses.

**Results:**

The study included 166 cases for which all NEW interview sheets for all periods were available. NEW interview sheets and stepwise screening tools during the first and second trimester were developed in which 2.5% of pregnant women were identified as requiring social support, respectively. The factors associated with EPDS ≧ 9 were “Women who felt confused/troubled or did not feel anything to be pregnant” (adjusted odds ratio [aOR]: 6.51, 95% conﬁdence interval [CI]: 1.62–26.15), “Maternal mental disorder” (aOR 4.38; CI 1.06–18.10), “Consultation request at first visit” (aOR 3.22; CI 1.09–9.45), and “Women who have difficulty or anxiety about pregnancy during the second trimester” (aOR 3.14; CI 1.29–7.67).

**Conclusions:**

We created the NEW interview sheets and screening tools during the first and the second trimester. Future studies are needed to validate these screening tools.

## Introduction

Social problems during pregnancy and postpartum were important risk factors for child maltreatment (1, 2). Child maltreatment causes major public health problems not only in childhood but also until adulthood (3–5). Among the verified child abuse cases which resulted in death (6), children under 1 year of age accounted for about 50% of deaths due to childhood maltreatment, and half of them (25%) are within the first month of life. Previously reported background factors related to child maltreatment include unexpected/unplanned pregnancies, failure to receive pregnancy health checkups, failure to receive the Maternal and Child Health Handbook, and teenage pregnancies (6, 7). Other factors, such as unmarried mothers, maternal mental disorder, low educational achievement, a history of childhood abuse, deprivation, and low-birth-weight children have also been reported in association with child maltreatment (2, 7, 8). Therefore, early preventive efforts against child maltreatment taken during pregnancy and the postnatal period are extremely important. Identification of at-risk parents such as mother without social support, maternal mental illness, and teenager during pregnancy and early intervention resulted in a decrease in the rate of referrals to child protective centers (9). Understanding social and medical risk factors is an essential part of abuse prevention. Identifying social risk factors at the time of pregnancy and initiating support for pregnant women who need social support may help reduce child maltreatment.

Although there are many public health centers that identify pregnant women who need social support through questionnaires and interviews when issuing the Maternal and Child Health Handbook, success is limited due to few opportunities for contact with pregnant women. In Japan, pregnancy health checkups at obstetric institutions are scheduled at least 14 times, where the mother's health condition is assessed, and a medical examination and health guidance are provided. Health guidance addresses the mental health of pregnant women and relieving their anxiety about pregnancy, childbirth, and childcare. However, about half of the delivery facilities in Japan are private obstetric clinics, some of which are inadequate for resolving social and psychological problems in the affected population. This study aimed to develop a screening tool for detecting pregnant women in need of support that can be used in health guidance at all facilities, including private obstetric clinics. We investigated the items from the NEW interview sheets that were considered important by women identified to be in need of social support. The primary outcome was to develop an available interview sheet during pregnancy and the screening tool to detect pregnant women in need of support in collaboration with a public health center. A secondary outcome was to investigate the perinatal factors associated with EPDS.

## Materials and methods

### Study design

This was a prospective cohort study conducted at three medical institutions that were familiar with social issues such as unmarried mothers, poverty, maternal mental disorder or teenage pregnancies affecting pregnancy and childcare outcomes. Inclusion criteria in the study were pregnant women managed at the three medical institutions who consented to the study. There were no exclusion criteria.

### Questionnaire and study procedures

We implemented the new screening questionnaire (NEW interview sheets) at the first visit, at approximately 28 weeks gestation, 36 weeks gestation, and the postpartum period. Table 1 provides an itemized list of question on the NEW interview sheets for each period. According to previous studies, variables to be used in the interview sheet were selected (2, 7, 10). These interviews were conducted at three medical institutions (Osaka Women's and Children's Hospital, Seibo Hospital, and Showa University Hospital) that actively identify pregnant women in need of support in collaboration with a public health center. In addition, the Edinburgh postnatal depression scale (EPDS) was administered together at one-month postpartum. We defined suspected postpartum depression as having an EPDS score of 9 or higher based on the results of a previous community study in Japan (11).

**Table 1:** NEW interview sheets for each period.

**First visit**	**Positive**	**Negative**
Maternal feelings toward being pregnant	Happy	Confused, Trouble, Did not feel anything to be pregnant
Partner's feelings toward being pregnant	Happy	Confused, Trouble, Did not feel anything to be pregnant
Depression symptoms	Nothing/Very few	Sometimes/Often
Family and social support	Yes	No
Economic status	No problem	Deprived/Need of public assistance
Partner's status	Married	Unmarried/Remarried
Maternal mental disorder	No	Yes
Illegal drug use by the mother	No	Yes
Illegal drug use by the partner	No	Yes
Worries about older child	No	Yes
Consultation request	No	Yes
Number of fetuses	Singleton pregnancies	Multiple pregnancies
Number of children (Exclusive of this pregnancy)	<3	≧3
Maternal age	<25	≧25
**Second trimester**	**Positive**	**Negative**
Maternity life	Almost happy	Difficulty/Anxiety
Talking with partner	Very often/Sometimes	Very few/Nothing
Partner violence	No	Yes
Worries about older child	No	Yes
Parent's own childhood abuse	No	Yes
Depression symptoms	Nothing/Very few	Sometimes/Often
Family and social support	Yes	No
Smoking	No/Discontinued after pregnant	Continued after pregnant
Alcohol use	No/Discontinued after pregnant	Continued after pregnant
Consultation request	No	Yes
Status of pregnancy health checkups	Usual	Less/Unscheduled
**Third trimester**	**Positive**	**Negative**
Depression symptoms	Nothing/Very few	Sometimes/Often
Worries about older child	No	Yes
Concerns about the course of pregnancy	No	Yes
Family and social support	Yes	No
Childbirth preparation	Almost finished	Unfinished
Consultation request	No	Yes
Pregnancy health checkups	Usual	Less/Unscheduled
Fetal congenital disease or fetal growth restriction	No	Yes
**Postpartum**	**Positive**	**Negative**
Fetal condition after childbirth	No	Yes
Feelings toward childcare	Happy	Neither/Unhappy
Partner's support	Often/Sometimes	Few
Family and social support	Yes	No
Difficulty of childcare	Nothing/Sometimes	Often
Consultation request	No	Yes
EPDS	<9	≧9

EPDS; the Edinburgh postnatal depression scale.

At the three medical institutions, information was collected from face-to-face interviews, impressions based on behavior and actions such as poor comprehension, disheveled clothing, medical information and original questionnaires including the NEW interview sheets were distributed. Medical information included maternal age, parity, medical history, maternal mental disorder, gestational age at the first visit, number of pregnancy medical examinations, multiple pregnancies, and pregnancy complications. Using information other than the NEW interview sheets, pregnant women in need of administrative cooperation were selected by conferences at Osaka Women's and Children's Hospital and Seibo Hospital and by nurses specializing in maternity nursing at Showa University. The members of the conference at Osaka Wemen's and Children's Hospitalwere nurses, midwives, public health nurses, caseworkers, and obstetricians, while the members at Seibo Hospital were nurses, midwives, caseworkers, and pediatricians.

### Statistical analyses

Standardized partial regression coefficients were calculated by multiple linear regression analysis to refine and score each item on the NEW interview sheet. Scores were calculated by multiplying the standardized partial regression coefficient by 100 and rounding it off to the closest whole. The cutoff value was calculated by the receiver operating characteristic (ROC) curve. The variance inflation factor was calculated using regression analysis to confirm multi-collinearity in the multivariate analysis. The association between perinatal factors and EPDS was assessed using multiple logistic regression analysis. All statistical analyses were performed using SPSS Statistics ver. 27 software. Two-sided *P*-values of <0.05 were considered statistically significant.

### Ethical statement

This study complied with all the relevant national regulations, institutional policies, and the tenets of the Helsinki Declaration. This research was approved by the Ethics Review Board of the University of Yamanashi (approval number 1663, approval date July 10, 2017). Written informed consent was obtained from all study subjects. At the time the NEW interview sheets from the three medical institutions were mailed to the University of Yamanashi, the data center, research IDs were assigned, and names and IDs for each hospital were deleted. The matching forms of research IDs and hospital-specific IDs were managed at each institution.

## Results

**Figure 1:**
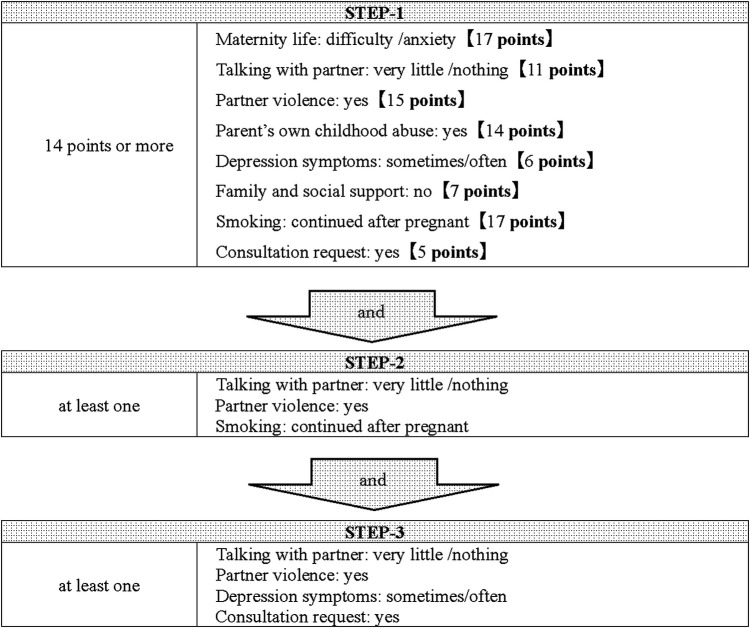
Support status change during pregnancy and the postpartum period. There were cases in which the support status changed during the gestational period. ^†^Hospital support: socially high-risk pregnant women who were supported by their respective obstetric institutions. ^‡^Administrative cooperation: pregnant women in need of support to require collaboration with public health center.

Table 2 illustrates the number of responses to the interview sheet and the number of pregnant women in need of social support who were judged to need cooperation with the public health center at each of the periods. We received 678 responses at the first visit, 495 during the second trimester, 296 during the third trimester, and 822 for postpartum. Figure 1 showed that the change in support status during pregnancy and postpartum for the 166 cases for which all NEW interview sheets for all periods were available. In this study, we investigated socially high-risk pregnant women who were supported by their respective obstetric institutions as well as collaborating with a public health center. In the first trimester, 2% of pregnant women in need of support required collaboration with a public health center and 17% of the pregnant women were at high-risk of needing social support. During the second and third trimesters, 6% of pregnant women in need of support required collaboration with public health centers, higher than that in early pregnancy, and it was highest at 23% in the postpartum period. There was a change in the support status during pregnancy. In some cases, cooperation with public health centers was started but later changed to support at the respective medical institutions or some cases in which support was no longer needed. There were also 13 cases presenting without problems during pregnancy, but support was needed after delivery.

**Table 2:** The number of pregnant women in need of support by the public health center.

	Osaka^c^	Seibo^d^	Showa^e^	Total
First visit				
Number of cases	279	120	277	678
Missing data	7	0	2	9
Social high-risk pregnant women^a^	41 (15)	5 (4)	67 (24)	113 (17)
Women in need of social support^b^	8 (3)	0	6 (2)	14 (2)
Second trimester				
Number of cases	225	46	224	495
Missing data	10	1	0	11
Social high-risk pregnant women^a^	23 (11)	3 (7)	8 (4)	34 (7)
Women in need of social support^b^	9 (4)	0	21 (9)	30 (6)
Third trimester				
Number of cases	171	40	85	296
Missing data	13	0	1	14
Social high-risk pregnant women^a^	17 (11)	2 (5)	18 (12)	37 (13)
Women in need of social support^b^	8 (5)	0	10 (12)	18 (6)
Postpartum				
Number of cases	199	61	562	822
Missing data	8	1	294	303
Social high-risk pregnant women^a^	5 (3)	4 (7)	99 (38)	108 (21)
Women in need of social support^b^	37 (19)	2 (3)	80 (30)	119 (23)

Data are shown as *n* ().

^a^ social high-risk pregnant women: pregnant women with social problems supported by their respective obstetric institutions.

^b^ women in need of social support: pregnant women in need of social support who were judged to need cooperation with the public health center at each of the periods.

^c^ Osaka: Osaka Women's and Children's Hospital.

^d^ Seibo: Seibo Hospital.

^e^ Showa: Showa University Hospital.

**Figure 2:**
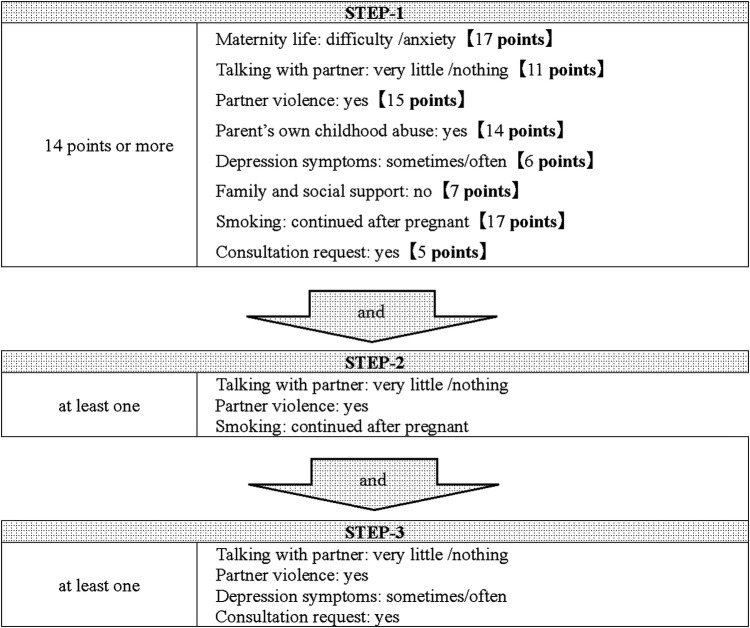
The screening tool used at the first visit. Pregnant women with score of 22 points or more were selected as STEP-1. Then, pregnant women are narrowed down as STEP 2 and 3 according to the items on the interview sheet at first visit.

Table 3 shows the results of the multiple linear regression analysis at the first visit. Compared to the results of the single regression analysis (Model-1) for each item, in the multiple regression analysis in which all factors were input, the results of the items “partner's feelings toward being pregnant “and “consultation request “were reversed, so they were excluded from the study. Furthermore, among the items on the interview sheet, “illegal drug use by the mother and partner” were excluded from the screening tool because they clearly required cooperation with a public health center. The questionnaire after exclusion of the four items was used as Model-2, and the score of each item was calculated. The cutoff value for the detection of pregnant women in need of social support was calculated to be 22 points from the ROC curve, and scores of 22 points or more were selected as STEP-1, with an area under the curve (AUC) of 0.96, the sensitivity of 0.92, and specificity of 0.92. Of the 558 subjects analyzed at the first visit, 54 cases were detected for STEP-1. To further improve the selection rate, we checked the applicable percentage of each item including the four items excluded in STEP-1 in the group that had cooperation with a public health center in this study. The factors that accounted for more than 30% of the total were “no family and social support,” “maternal mental disorder,” “worry about the older child,” and “multiple pregnancies,” and those who had at least one of these four factors were selected as STEP-2 in addition to STEP-1. As a result, 20 cases were selected, of which six cases (30%) were involved in cooperation with a public health center. In addition, those with at least two of the following were selected as STEP-3: “depression symptoms,” “maternal mental disorder,” and “consultation request.” The number of pregnant women in need of social support based on the screening by three STEPs was 14, of which 5 (36%) required administrative cooperation. The number of positive screening results was 14 (2.5%) out of 558. The screening tool is presented in Figure 2.

**Table 3:** The multiple linear regression analysis at first visit.

	Model-1 (clude)			Model-2 (adjusted; *n* = 554)					
Items	OR	95% CI	*P*	SPRC	Score	OR	95% CI	*P*	VIF
Maternal feelings toward being pregnant									
Happy	1	Reference				1	Reference		
Unhappy^a^	10.985	3.966–30.422	0	0.069	6.9	2.72	0.509–14.527	0.242	1.095
Partner's feelings toward being pregnant	–								
Happy	1	Reference							
Unhappy^a^	5.566	1.723–17.98	0.004						
Depression symptoms									
No	1	Reference				1	Reference		
Yes	3.987	1.135–14.005	0.031	0.019	1.9	1.794	0.312–10.298	0.512	1.058
Family and social support									
Yes	1	Reference				1	Reference		
No	13.489	1.325–137.317	0.028	0.117	11.7	32.455	1.386–759.856	0.031	1.059
Economic status									
No problem	1	Reference				1	Reference		
Poverty	10.235	3.787–27.66	0	0.076	7.6	2.24	0.473–10.613	0.31	1.167
Partner's status									
Married	1	Reference				1	Reference		
Unmarried/remarried	6.433	2.324–17.812	0	0.129	12.9	6.068	1.33–27.686	0.02	1.086
Maternal mental disorder									
No	1	Reference				1	Reference		
Yes	13.593	4.653–39.709	0	0.16	16	11.131	1.662–74.542	0.013	1.034
Illegal drug use by mother	–								
No	1	Reference							
Yes	0	0	0.999						
Illegal drug use by partner	–								
No	1	Reference							
Yes	0	0	0.999						
Worries about older child									
No	1	Reference				1	Reference		
Yes	2.154	0.685–6.767	0.189	0.028	2.8	2.917	0.367	0.312	1.02
Consultation request	–								
No	1	Reference							
Yes	3.689	1.322–10.296	0.013						
Number of fetuses									
Singleton	1	Reference				1	Reference		
Multiple	4.96	1.049	0.043	0.105	10.5	14.601	1.557	0.019	1.01
Number of children									
<3	1	Reference				1	Reference		
≧3	7.239	1.903	0.004	0.141	14.1	19.624	2.45	0.005	1.103
Maternal age									
≧25	1	Reference				1	Reference		
<25	15.49	4.909	0	0.212	21.2	37.134	5.458	0	1.039

OR: odds ratio.

95% CI: 95% confidential interval.

VIF: variance inflation factor.

SPRC: standardized partial regression coefficient.

^a^ Unhappy: confused, trouble, did not feel anything to be pregnant.

**Figure 3:**
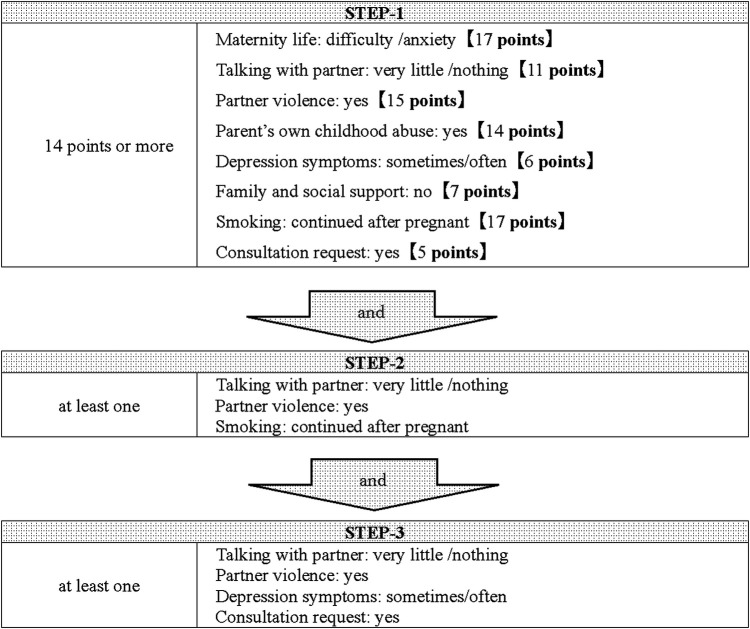
The screening tool used during the second trimester. Pregnant women with score of 14 points or more were selected as STEP-1. Then, pregnant women are narrowed down as STEP 2 and 3 according to the items on the interview sheet during the second trimester.

Table 4 shows the results of the multiple linear regression analysis during the second trimester. Compared to the results of the single regression analysis (Model-1) for each item, in the multiple regression analysis in which all factors were entered, the item “worries about the older child “was reversed, so it was excluded from the study. In addition, “alcohol use” and “status of pregnancy health checkups “were excluded because they could not be analyzed due to missing data. The NEW interview sheets after the exclusion of the three items were used as Model-2, and the scores for each item were calculated. The cutoff value for pregnant women in need of social support was determined to be 14 points from the ROC curve, and scores of 14 points or more were extracted as STEP-1, with an AUC of 0.77, sensitivity of 0.82, and specificity of 0.62. Of the 483 cases analyzed during the second trimester, 110 cases were extracted by STEP-1, of which 34 cases (31%) were had cooperation with public health centers in this study. To increase the selection rate, we examined the factors on the NEW interview sheets of the women who scored more than 14 points. Since the percentage of the risk group was more than 30% for all items, we calculated the Spearman correlation coefficient for the subjects with 14 points or more, weighted the correlation coefficient by a factor of 10, and considered variables with 2 points or more to be factors with higher risk. These items were “no talking with partner,” “partner violence,” and “smoking,” and if any of these items were applicable, they were narrowed down as STEP-2 in addition to STEP-1. Twenty-one people were selected for STEP-1 and STEP-2, of whom 12 (57%) required administrative cooperation in this study. In addition, STEP-3 was selected for those who had at least one of the following: “no talking with partner,” “partner violence,” “depression symptoms,” and “consultation request.” As a result, 12 cases were selected, and all of them were the subjects of the actual administrative collaboration in this study. The number of positive cases by the screening by three STEPs was 12 out of 483 (2.5%). The screening tool is shown in Figure 3.

**Table 4:** The multiple linear regression analysis during the second trimester.

	Model-1 (clude)			Model-2 (adjusted) (*n* = 472)					
Items	OR	95% CI	*P*	SPRC	score	OR	95% CI	*P*	VIF
Maternity life									
Happy	1	reference				1	Reference		
Difficulty/Anxiety	4.165	2.306–7.525	0	0.167	17	3.19	1.603–6.35	0.001	1.119
Talking with partner									
Yes	1	Reference				1	Reference		
No	6.773	2.413–19.008	0	0.108	11	3.102	0.819–11.752	0.096	1.233
Partner violence									
No	1	Reference				1	Reference		
Yes	12.918	3.523–47.367	0	0.15	15	7.081	1.502–33.388	0.013	1.236
Worries about older child									
No	1	Reference							
Yes	1.342	0.598–3.009	0.476						
Parent's own childhood abuse									
No	1	Reference				1	Reference		
Yes	6.413	2.695–15.259	0	0.135	14	3.566	1.281–9.929	0.015	1.083
Depression symptoms									
No	1	Reference				1	Reference		
Yes	2.74	1.547–4.852	0.001	0.058	6	1.531	0.785–2.984	0.211	1.114
Family and social support									
Yes	1	Reference				1	Reference		
No	7.691	0.474–124.717	0.151	0.072	7	7.517	0.407–138.934	0.175	1.004
Smoking									
No	1	Reference				1	Reference		
Yes	24.113	2.464–236.008	0.006	0.165	17	26.667	2.316–306.98	0.008	1.019
Alcohol use									
No	1	Reference							
Yes	0	0	0.999						
Consultation request									
No	1	Reference				1	Reference		
Yes	2.051	1.158–3.632	0.014	0.053	5	1.499	0.78–2.881	0.225	1.064
Pregnancy health checkups									
Usual	1	reference							
Less/unscheduled	125,419,59234	0	1						

OR: odds ratio.

95% CI: 95% Confidential Interval.

VIF: Variance Inflation Factor.

SPRC: standardized partial regression coefficient.

Table 5 shows the results of the multiple linear regression analysis during the third trimester. Each item on the NEW interview sheets was scored, and a cutoff value of 7 points was calculated using the ROC curve. The AUC, sensitivity, and specificity were 0.46, 0.43, and 0.57, respectively, which were inappropriate for screening.

**Table 5:** The multiple linear regression analysis during the third trimester.

	Model-1 (clude)			Model-2 (adjusted) (*n* = 296)				
Items	OR	95% CI	*P*	SPRC	score	OR	95% CI	*P*
Depression symptoms								
No	1	Reference				1	Reference	
Yes	2.329	0.617–8.793	0.2123	0.288	29	2.865	0.54–15.204	0.2165
Worries about older child								
No	1	Reference				1	Reference	
Yes	3.039	0.872–10.591	0.0809	0.1226	12	1.88	0.344–10.28	0.4664
Concerns about the course of pregnancy								
No	1	Reference						
Yes	2.483	0.73–8.446	0.1453					
Family and social support								
Yes	1	Reference			28	1	Reference	
No	53.397	4.462–638.95	0.0017	0.276		124.973	3.724–>999.9	0.0071
Childbirth preparation								
Finished	1	Reference				1	Reference	
Unfinished	3.416	0.386–30.227	0.2695	0.0653	7	2.027	0.144–28.527	0.6006
Consultation request								
No	1	Reference				1	Reference	
Yes	4.458	1.306–15.218	0.017	0.5232	52	7.603	1.54–37.545	0.0128
Pregnancy health checkups								
Usual	1	Reference				1	Reference	
Less/unscheduled	12.182	1.025–144.745	0.0477	0.2416	24	68.484	3.034–>999.9	0.0079
Fetal congenital disease or fetal growth restriction								
No	1	Reference				1	Reference	
Yes	7	1.923–25.48	0.0032	0.4354	44	18.455	3.628–93.887	0.0004

OR: odds ratio.

95% CI: 95% Confidential Interval.

VIF: Variance Inflation Factor.

SPRC: standardized partial regression coefficient.

In addition, we examined the relationship between the EPDS at the one-month postpartum and each item on the NEW interview sheets. Of those who completed the NEW interview sheets at the first visit, during second trimester, and during third trimester, and third trimester, 415 (66.7%), 384 (77.6%), and 232 (78.4%), respectively, underwent postpartum EPDS. Of these, 28 (6.7%) at the first visit, 24 (6.3%) during second trimester, and 17 (7.3%) during third trimester had an EPDS score of 9 or higher. Table 6 showed the relationship between EPDS ≧ 9 and each item on the NEW interview sheets. The factor “no family and social support” (*n* = 1) and “ illegal drug use by the mother(*n* = 4) and partner” (*n* = 3) at the first visit, and “no family and social support,”(*n* = 2) “alcohol use,” (*n* = 2) and “less or unscheduled pregnancy health checkups”(*n* = 1) during the second trimester were excluded due to their small number. The factors associated with EPDS ≧ 9 were “Women who felt confused/troubled or did not feel anything to be pregnant” (adjusted odds ratio [aOR]: 6.51, 95% conﬁdence interval [CI]: 1.62–26.15), “Maternal mental disorder”(aOR 4.38, CI 106–18.10), “Consultation request at first visit” (aOR 3.22, CI 1.09–9.45), and “Women who have difficulty or anxiety about pregnancy during the second trimester” (aOR 3.14, CI 1.29–7.67). The factor “less or unscheduled pregnancy health checkups” (*n* = 3) during the third trimester was excluded due to the small number of cases, and no factor associated with EPDS was found.

**Table 6:** Relationship between EPDS ≧ 9 and each item on the NEW interview sheets.

	cOR	95% CI	*P*	aOR	95% CI	*P*
At first visit						
Maternal feelings (confused/troubled or did not feel anything)	4.62	1.80–11.86	0.002	6.51	1.62–26.14	0.008
Partner's feelings (confused/troubled or did not feel anything)	1.46	0.32–6.6	0.62	0.35	0.03–3.51	0.37
Depression symptoms	2.75	1.19–7.11	0.02	2.86	0.75–10.92	0.13
Poverty	1.9	0.74–4.93	0.19	1.46	0.37–5.72	0.58
Unmarried/remarried	1.25	0.42–3.77	0.69	2.44	0.61–9.69	0.21
Maternal mental disorder	5.92	1.95–18.00	0.002	4.38	1.06–18.10	0.04
Worries about older child	1.43	0.52–3.92	0.49	1.41	0.33–6.14	0.64
Consultation request	3.31	1.47–7.43	0.004	3.22	1.09–9.45	0.03
Multiple pregnancy	3.18	0.65–15.48	0.15	1.97	0.19–20.43	0.57
Number of children ≧3	1	0.13–7.91	1	0.85	0.05–13.43	0.91
Maternal age <25	1.06	0.06–5.62	0.96	0.8	0.06–10.22	0.86
During the second trimester						
Matanity life (Difficulty/Anxiety)	4.18	1.81–10.02	0.0009	3.15	1.29–7.67	0.01
Talking with partner	2.63	0.55–12.48	0.22	1.76	0.27–11.41	0.56
Partner violence	1.91	0.23–15.91	0.55	1.12	0.09–13.48	0.93
Worries about older child	1.78	0.64–5.01	0.27	1.14	0.37–3.52	0.82
Parent's own childhood abuse	1.95	0.42–9.04	0.39	0.77	0.13–4.42	0.77
Depression symptoms	3.11	1.3–7.45	0.01	2.42	0.96–6.10	0.06
Smoking	5.17	0.52–51.71	0.16	5.85	0.50–68.9	0.16
Consultation request	2.5	1.08–5.76	0.03	1.96	0.80–4.80	0.14
During the third trimester						
Depression symptoms	3.36	0.94–12.04	0.06	4.65	0.94–22.94	0.06
Worries about older child	1.68	0.45–5.08	0.41	1.7	0.41–7.10	0.46
Concerns about the course of pregnancy	2.36	0.83–6.73	0.11	2.01	0.54–7.54	0.3
Family and social support	13.25	0.79–221.8	0.07	24.14	0.56–1035.66	0.1
Childbirth preparation	0.96	0.26–2.86	0.95	0.33	0.066–1.69	0.18
Consultation request	2.49	0.89–6.94	0.08	1.71	0.48–6.09	0.41
Fetal congenital disease or fetal growth restriction	1.66	0.25–6.61	0.55	0.56	0.06–5.24	0.62

OR: odds ratio.

95% CI: 95% Confidential Interval.

## Discussion

The NEW interview sheets and the stepwise screening tool by three STEPs tools during the first and second trimester were developed in which 2.5% of pregnant women were identified as requiring social support, respectively. The screening tool was created with the intention of narrowing down the number of targets because if there are too many targets, the health center may not be able to handle them. According to a survey in Osaka Prefecture, cases of deliveries of little or no receiving for pregnancy health checkups accounted for about 0.3%. (Unpublished data) In addition, the frequency of specified expectant mothers was reported to be 2%–5% (12, 13). Specified expectant mothers are defined in the Child Welfare Act as “pregnant women who are recognized as being in particular need of support during pregnancy with regard to postpartum care.” A specified expectant mother is determined by the health center. This study aims to detect pregnant women in need of support in collaboration with health centers, that is, cases corresponding to specified pregnant women. Therefore, we believe that the detection rate of pregnant women in need of social support using this screening tool is reasonable. The screening tool was created with the goal of being able to detect women, even in obstetric clinics that were not familiar with social issues, automatically. In the future, it will be necessary to verify whether these NEW interview sheets and screening tools can appropriately identify pregnant women in need of social support, and we are planning to create a system that can automatically identify pregnant women in need of social support by inputting the results of the NEW interview sheets.

The frequency of postpartum depression is reported to be 5%–10% in a prospective study in Japan (14) and 9% in the final report of the “Healthy Parents and Children 21” (15). Furthermore, postpartum depression may lead to maternal suicide as well as child maltreatment (16–18). In Japan, a suicide prevention program (Nagano model) (19) has been developed to improve postpartum mental health and reduce maternal suicide, and its effectiveness has been reported. In the present study, the factors associated with EPDS ≧ 9 at one-month postpartum were “Women who felt confused/troubled or did not feel anything to be pregnant,” “Maternal mental disorder,” “Consultation request at first visit,” and “Women who have difficulty or anxiety about pregnancy during the second trimester.” Although postpartum depression may develop unexpectedly, our findings have been suggested that conditions that could cause postpartum depression existed during pregnancy. In addition, it suggested that identifying mothers who have mental disorder, unwanted pregnancy, and anxiety during pregnancy may be useful in early detecting women who have postpartum depression. According to the reports of the Japan Environment and Children's Study, compared with women who felt very happy to be pregnant, those whose pregnancy was unintended but happy, unintended, and confused, those who felt troubled, and those who felt no emotion toward being pregnant had increased risks of postpartum depression (20).

The strength of this study is a prospective survey conducted in several obstetric institutions that are specialized in addressing social issues, and various criteria were used to determine the detection of pregnant women in need of social support. In addition, considering the possibility of changes in the mother's and family's condition during pregnancy and background factors that may become apparent through the establishment of a relationship between the mother and the obstetric institution, NEW interview sheets and screening tools were developed that included items tailored to each period and items common to all periods.

Several limitations should be addressed. First, less than half of the cases had a complete NEW interview sheets for all time periods. This was because two of the three facilities in this study handle many medically high-risk pregnancies, which may result in first visits at various times. In those facilities, the number of patients who were referred in the second trimester and later due to medical risks such as fetal growth retardation or threatened premature birth means that a larger percentage of patients do not receive the NEW interview sheets, especially in the early and middle trimester of pregnancy. Second, although it had been reported that a late first visit is associated with pregnant women in need of social support, it was not included in this study. This was because the study includes many cases in which the first visit was made after the second trimester due to medical risks. Finally, this study was conducted up to one month after childbirth, and the relationship with the need for support at a public health center after that was unknown. Although it would be desirable to collate the data with information from the public health center if there was an actual fear of child maltreatment several months after the birth, it was difficult to obtain the data from the public health center from the viewpoint of personal information protection. In a survey such as this one, it is considered necessary to create an environment where medical institutions and government agencies can collaborate to collect information in Japan.

In conclusion, we developed the NEW interview sheets and the stepwise screening tools during the first and the second trimester to detect pregnant women in need of support to require collaboration with a public health center. We plan to further validate these NEW interview sheets and screening tools by increasing the number of participating obstetrics clinics.

## Data Availability

The raw data supporting the conclusions of this article will be made available by the authors, without undue reservation.
